# Maximum Strength Benchmarks for Difficult Static Elements on Rings in Male Elite Gymnastics

**DOI:** 10.3390/sports9060078

**Published:** 2021-05-28

**Authors:** Christoph Schärer, Sarina Huber, Pascal Bucher, Claudio Capelli, Klaus Hübner

**Affiliations:** Department of Elite Sport, Swiss Federal Institute of Sport Magglingen (SFISM), 2532 Magglingen, BE, Switzerland; sarina.h77@hotmail.com (S.H.); bucher.pascal@bluewin.ch (P.B.); claudio.capelli@stv-fsg.ch (C.C.); klaus.huebner@baspo.admin.ch (K.H.)

**Keywords:** maximum strength, eccentric, concentric, isokinetic, isoinertial, training, artistic gymnastics, rings

## Abstract

On rings, in men’s artistic gymnastics, the general strength requirements for important static elements remain elusive. Therefore, the aim was to describe the relationship between a new conditioning strength test and a maximum strength test of static elements on rings in order to determine the minimal strength level (benchmarks) required to maintain these elements with one’s own body weight. Nineteen elite gymnasts performed a concentric (1RM isoinertial) and eccentric (isokinetic: 0.1 m/s) conditioning strength test for swallow/support scale (supine position) and inverted cross (seated position) on a computer-controlled device and a maximum strength test maintaining these elements for 5 s on rings with counterweight or additional weight. High correlation coefficients were found between the conditioning maximum strength for swallow/support scale (r: 0.65 to 0.92; *p* < 0.05) and inverted cross (r: 0.62 to 0.69; *p* > 0.05) and the maximum strength of the elements on rings. Strength benchmarks varied between 56.66% (inverted cross concentric) and 94.10% (swallow eccentric) of body weight. Differences in biomechanical characteristics and technical requirements of strength elements on rings may (inter alia) explain the differences between correlations. Benchmarks of conditioning strength may help coaches and athletes systematize the training of strength elements on rings.

## 1. Introduction

**Figure 1:**
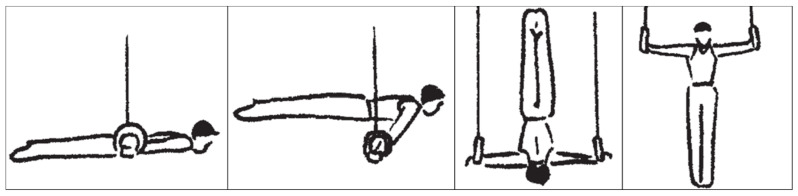
Important strength elements on rings. The most frequently performed strength elements in competition routines of elite gymnasts (from left: swallow, support scale, inverted cross, iron cross [1]). These copyrighted drawings were provided courtesy of FIG/Koichi Endo and may not be reproduced or copied without written authorization of FIG.

With recent developments in men’s artistic gymnastics, strength elements have become the most important components of competition routines on rings. According to the Code of Points (CoP) [1], a routine on rings can include a maximum of eight strength elements, each of which must be maintained for at least two seconds. In order to execute these strength elements as technically cleanly as possible, a high level of relative maximum strength and advanced balance skills in the specific holding positions are crucial [2]. Among the permitted strength elements, the “swallow” is the most important element due to its high difficulty value [3]. Additionally, elite athletes frequently perform the elements support scale, inverted cross, and iron cross, although these have slightly lower difficulty values (Figure 1).

To develop the required ring-specific maximum strength in training, athletes have traditionally employed concentric or static barbell or dumbbell exercises for the relevant muscle groups or facilitated versions of the elements themselves, such as with a counterweight, a device that shortens the lever arm (herdos), or with a spotter [4,5,6,7]. However, previous studies have shown that not all of these approaches provoke similar muscle activation patterns as the elements themselves [5,6,8], and that the maximum strength in these traditional exercises is not always closely related to the ring-specific strength performing the elements [4,7]. Recently, eccentric exercises have been successfully included into strength training in order to simulate the decelerating muscle work (to overcome gravity) when holding a strength element [2]. Nevertheless, both concentric and eccentric exercises are important for developing the required strength and to prepare the athletes’ upper-body muscles for the strain of the static strength elements on rings. In order to plan and monitor an efficient strength training program, tests must be established to assess ring-specific strength as well as the level of strength for specific conditioning exercises. Previous studies focused on measuring strength on rings using force plates [9,10] or either a counterweight or additional weight [2,4,11]. Although strength could be assessed accurately [9], investigators deemed it nonetheless difficult to derive specific training recommendations from their test results. In this context, combining conditioning strength tests with ring specific strength tests could provide important information about how much conditioning strength an athlete gained and how an athlete is able to apply this strength when performing strength elements on rings. This could help direct training on either developing more conditioning strength or on learning how to apply the force during hold elements (“technical” strength training of the static positions on rings). Furthermore, the effectiveness of the different training phases for improving conditioning and/or ring specific strength could be monitored more easily.

Recently, computer-controlled training devices have been developed, which allow force, velocity, and power to be monitored during strength training and testing. The different movement modalities (eccentric, concentric, isokinetic, and isoinertial), a variety of stimuli, and the flexible cable pulley system allow the development of new (computer controlled) specific training exercises and tests. In a recent study [2], a highly effective new gymnastic-specific eccentric–isokinetic strength exercise was developed with the help of such a device. Based on these results, two new conditioning, gymnastic-specific, concentric, and eccentric maximum strength tests for the elements swallow/support scale and inverted cross were developed using the same computer-controlled device.

Therefore, the first aim of this study was to calculate the relationship between the conditioning maximum strength test values (concentric and eccentric) and the maximum strength performing the static elements swallow, support scale, and inverted cross on rings. The second aim was to determine conditioning maximum concentric and eccentric strength benchmarks needed to maintain these strength elements on rings with one’s own body weight.

## 2. Materials and Methods

Nineteen male international (*n* = 9) and national (*n* = 10) elite gymnasts (age: 22.03 ± 2.5 years; weight: 64.99 ± 5.27 kg; height: 169.38 ± 4.81 cm) volunteered to participate in this study. All athletes were in a good health and followed a similar training program (composed by the head coach of the national team) with a training volume of more than 25 h per week. All participating athletes were informed about the test procedures and gave their written informed consent to take part. The study was approved by the local ethics committee (project-ID: 2018-00742) and conducted in accordance with the current version of the Declaration of Helsinki, the guidelines for good clinical practice (ICH-GCP ISO EN 14155), and all national legal and regulatory requirements.

All tests were conducted on the same day for a given athlete. First, maximum strength performing the elements swallow, support scale or inverted cross on rings was determined, and then, the conditioning maximum concentric and eccentric strength was assessed using a computer-controlled device (1080 Quantum Syncro, 1080 Motion, Lindingö, Sweden). Athletes had at least two hours of rest in between ring-specific and conditioning maximum strength tests.

### 2.1. Maximum Strength Test Ring Elements

**Figure 2:**
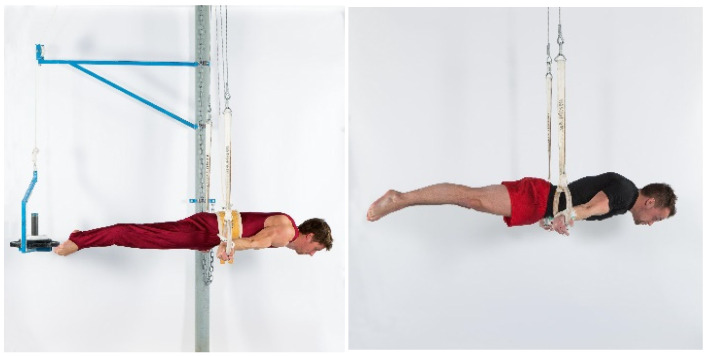
Maximum strength test swallow on rings. Maximum strength test of the element swallow on rings (holding time 5 s) with counterweight (**left**) and with additional weight (**right**).

**Figure 3:**
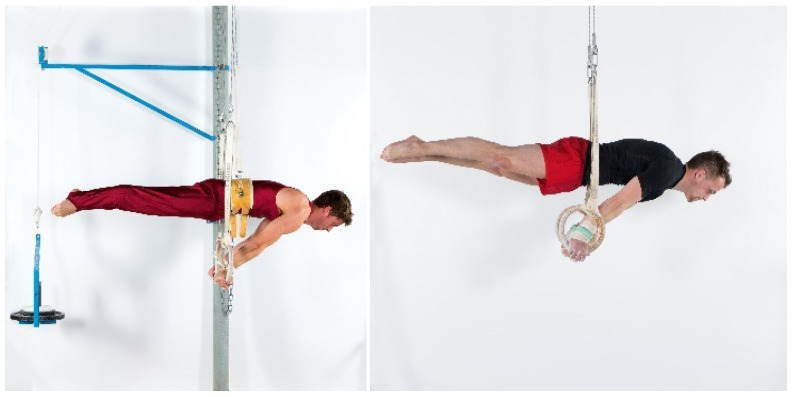
Maximum strength test of support scale on rings. Maximum strength test of the element support scale on rings (holding time 5 s) with counterweight (**left**) and with additional weight (**right**).

**Figure 4:**
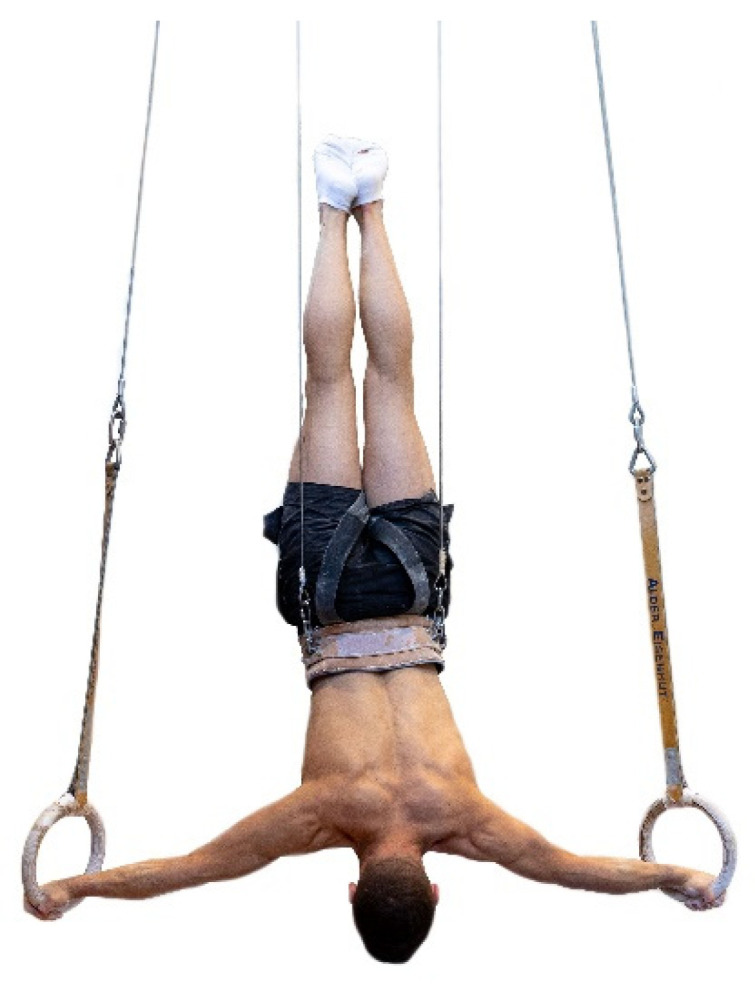
Maximum strength test of inverted cross on rings. Maximum strength test of the element inverted cross (holding time 5 s) with counterweight.

After an individual, gymnastic-specific warm-up lasting 20 min, maximum strength (defined as the maximal resistance that could be held for 5 s) for the elements swallow (Figure 2), support scale (Figure 3), and/or inverted cross (Figure 4) was assessed using either a pulley system (with counterweight) to facilitate the exercise or a weight belt to increase the resistance. The counterweight or additional weight was chosen dependent on the individual strength level of each gymnast in order to achieve exhaustion after exactly five seconds. Attempts were only valid if the required position (according to the Code of Points, less than 45° deviation of body angles from the perfect position [1]) could be maintained for the entire five seconds. The attempts filmed using an iPad (iPad Pro 9.7”, Apple Corporation, Cupertino, CA, USA). Holding time and holding positions (body angles) were verified with the video analysis software Dartfish (Dartfish SA, Fribourg, Switzerland).

### 2.2. Conditioning Maximum Concentric and Eccentric Strength Test

In order to assess conditioning maximal concentric and eccentric strength, two different conditioning exercises were used. One exercise involved similar muscle groups as when performing swallow and support scale and one exercise was similar to inverted cross. The exercises were performed on a computer-controlled training device (1080 Quantum Syncro), first concentrically and then eccentrically.

#### 2.2.1. Conditioning Strength Test for Swallow/Support Scale

**Figure 5:**
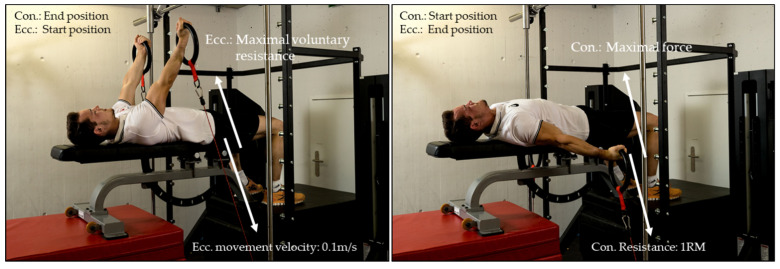
Conditioning strength test for swallow/support scale. Conditioning maximum concentric (con.) and eccentric (ecc.) strength test for the elements swallow and support scale on the computer-controlled device 1080 Quantum.

The assessment of the maximum concentric and eccentric conditioning strength for the elements swallow and support scale involved the gymnasts lying in a supine position on a bench with their arms extended and a shoulder angle of 50–70° (Figure 5). In their hands, they held a set of rings that were attached to synchronized cables of the computer-controlled device. For the assessment of the maximum concentric strength, the cables were first reeled in synchronously with a constant velocity of 0.2 m/s. The gymnasts followed this eccentric movement passively with stretched arms until the arms were in a horizontal position. At this moment, the examiner gave an acoustic sign and the gymnast pushed the rings, maintaining straight arms, with maximum speed and maximum force concentrically (and against the resistance of the device) back (upwards) to the initial position. The gymnasts performed four to five repetitions with increasing resistance (≈20% 1RM, ≈40% 1RM, ≈60% 1RM, ≈80% 1RM), starting with a low resistance and ending with the maximum resistance with which the athlete was able to perform only one repetition (1RM). Between the attempts, gymnasts had at least two minutes rest. After the assessment of the maximal concentric force, the athletes had 15 min rest before they performed the conditioning maximal eccentric strength test.

In order to assess the maximal eccentric strength, the starting position was the same as previously described for the measurement of the conditioning concentric strength (50°–70° shoulder angle, i.e., similar to the support scale position). In contrast to the execution of the concentric 1RM, the cables were reeled in synchronously with a constant velocity of 0.1 m/s, and the gymnasts provided maximal voluntary resistance to the rings with arms fully stretched until they reached a shoulder angle of approximately −15° to (below) horizontal position (i.e., similar to the position of swallow). Each gymnast had three attempts with at least two minutes rest in between.

#### 2.2.2. Conditioning Strength Test for Inverted Cross

**Figure 6:**
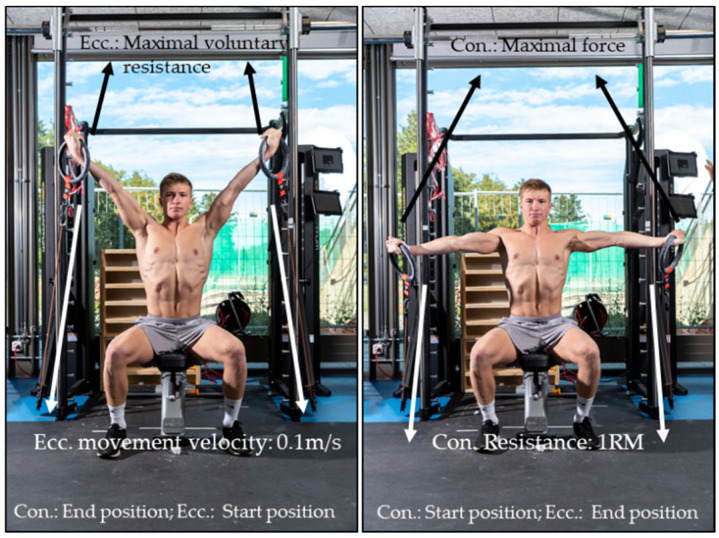
Conditioning strength test for inverted cross. Conditioning maximum concentric (con.) and eccentric (ecc.) strength test for the element inverted cross with the computer-controlled device 1080 Quantum.

In order to assess the maximum eccentric and concentric conditioning strength for the inverted cross, the procedures were similar to those described above for swallow/support scale, but with a different body position. Gymnasts sat in an upright position on a bench without any fixation of the back and with their arms in 45° vertical position (Figure 6).

For the assessment of concentric maximum strength, the cables were then reeled in with a constant velocity of 0.2 m/s and thereby lowered the gymnast’s arms passively in the sagittal plane to a horizontal position, at which point they pushed their arms (after a vocal signal) with maximum speed and force against the motor-controlled resistance back up to the starting position. Gymnasts performed several repetitions, starting with a low resistance and ending with 1RM.

The maximal eccentric strength was assessed similarly to the assessment for swallow and support scale. From the starting position (sitting upright, arms 45° vertical), maximal voluntary force was applied as the cables were reeled in at a constant velocity of 0.1 m/s until the arms reached a position of approximately −15° to (below) the horizontal. Each athlete had three attempts.

**Figure 7:**
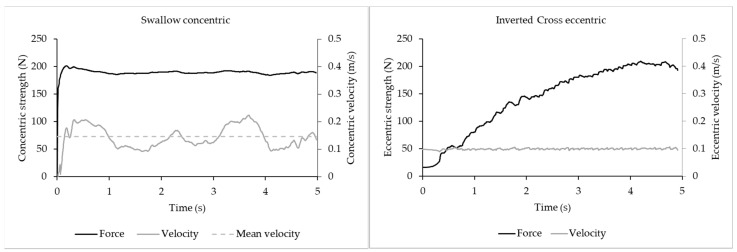
Force–velocity curves of conditioning tests. Typical concentric and eccentric force and velocity curves of the concentric and eccentric maximum strength tests (swallow and inverted cross) measured with the computer-controlled training device (1080 Quantum Syncro).

During each concentric and eccentric repetition, the device recorded the applied force for both left and right side (Figure 7). For each test, the duration of concentric and eccentric efforts were similar (~5 s) to those in the ring-specific strength tests. The attempt with the highest maximum concentric or eccentric force was used for the calculations in this study.

### 2.3. Statistical Analyses

Descriptive statistics were calculated and normal distribution was confirmed using the Kolmogorov–Smirnov test. Pearson’s correlation coefficient (r) was used to assess the relationships between the conditioning maximum strength tests and the maximum strength tests of ring elements. In order to calculate benchmark values for conditioning concentric and eccentric strength, linear regressions between each conditioning strength value (concentric and eccentric force, relative to body weight) coinciding ring-specific strength value (body weight + additional weight or − counterweight). The resulting regression equations were used to calculate the required minimal conditioning concentric and eccentric strength to perform the element body weight (without a counterweight). Test–retest reliability of the conditioning maximum concentric and eccentric strength tests for swallow/support scale and inverted cross was assessed from a total number of 34 and 10 eccentric and 32 and 10 concentric tests, respectively, which were performed within the same week. Eccentric tests displayed higher correlation coefficients (ICC > 0.95) than concentric tests (ICC = 0.86), but for concentric tests, smaller variation coefficients (CV%) were found (CV% < 3.34%) than for eccentric tests (CV% < 5.48%). Furthermore, no systematic errors were found (*t*-test: *p* > 0.05). These results agreed with previous studies that found high reliability using isokinetic [12] and isoinertial exercises modes [13] with the same computer-controlled device. The level of statistical significance was set to *p* < 0.05. The statistical analysis was performed using SPSS 22 software (SPSS, Inc., Chicago, IL, USA).

## 3. Results

All athletes completed the maximum strength tests on rings and the conditioning maximum concentric and eccentric strength tests on the training device. Individual results can be found in the Supplementary Material of this publication (Table S1). The mean values (± standard deviation) of these tests are displayed in Table 1. Four and seven athletes performed the elements swallow and support scale with body weight or additional weight, respectively. All athletes performed inverted cross with a counterweight. Individual results are displayed in Table S1 (Supplementary Material).

The relationships between the maximum strength for the elements swallow, support scale, and inverted cross on rings and the conditioning concentric and eccentric maximum strength tests for swallow/support scale and inverted cross are displayed in Table 2. Conditioning maximum concentric and eccentric strength explained 76% and 85% (R^2^) of the variability in maximum strength performing the element swallow, respectively. For the elements support scale (concentric: R^2^ = 0.59, eccentric: R^2^ = 0.42) and inverted cross (R^2^ = 0.48, *p* = 0.06, eccentric: R^2^ = 0.38, *p* = 0.10), these values were lower.

For all conditioning strength tests, eccentric Fmax was greater than concentric Fmax (by 25.56% to 27.81%). However, athletes with a higher ratio of eccentric-to-concentric strength tended to display greater ring specific strength; the individual eccentric-to-concentric strength ratio explained 37% of the variability in strength for swallow, but only 12% and 10% for support scale and inverted cross, respectively.

The conditioning concentric and eccentric strength benchmarks (values needed to hold the elements on rings with one’s own body weight) revealed that greater concentric and eccentric relative strength is required for the element swallow than for the elements support scale and inverted cross (Table 3).

## 4. Discussion

This study analyzed the relationships between two ring-specific conditioning maximum concentric and eccentric strength tests on a computer controlled training device and the maximum strength performing the elements swallow, support scale and inverted cross on rings. Furthermore, the conditioning concentric and eccentric strength benchmarks for maintaining the static elements swallow, support scale, and inverted cross on rings with one’s own body weight were calculated.

### 4.1. Relationships between Conditioning Maximum Strength Tests and Maximum Strength Tests on Rings

The results showed significant correlations (r: 0.65 to 0.92; *p* < 0.05) between the maximum strength performing the elements swallow and support scale and concentric and eccentric maximum conditioning strength. The correlations between conditioning strength values and the ring-specific strength for the element inverted cross (r: 0.62 to 0.69) were positive although non-significant. According to Hopkins [14], the magnitude of correlation coefficients can be interpreted as large to nearly perfect. Therefore, the concentric and eccentric conditioning strength tests in this study appear to be a valuable tool for determining the ring-specific strength. However, to better understand and interpret the observed relationships, differences between the three elements on rings, differences in test setups, and differences between the concentric and eccentric conditioning test will be discussed.

In order to maintain a strength element on rings, advanced balance skills enable the athlete to find the position in which the required force (to oppose gravity) can be applied to the unstable rings. However, from a biomechanical standpoint, the different holding positions impose different balance skill requirements. In this context, the holding position for the element swallow is more stable compared to the elements support scale and inverted cross. The body’s center of gravity is at a similar height as the rings, and the support is generally quite wide. Therefore, keeping balance is less of an issue, which makes it easier to apply one’s strength. This may explain why maximum strength performing the element swallow was explained almost completely (85%), and to the greatest degree among the investigated elements, by the conditioning maximum strength. For the elements support scale (up to 59%) and inverted cross (up to 48%) as well, conditioning strength is the primary prerequisite. However, because the body’s center of gravity is above the rings for these two elements, gymnasts must not only resist the downward force of gravity but also (from a subjective perspective) avoid falling forward and backwards (in particular for inverted cross) in order to hold the positions. This challenge is exacerbated by the relatively narrow basis of support for the support scale and the rather long lever arm of the body’s center of mass for the inverted cross. Feasibly, this situation impedes gymnasts’ ability to apply their full strength for these elements. At the very least, multi-directional force application and balance are decisive factors for the performance of these elements on the rings (more so than for swallow); however, these are not required by the conditioning strength test exercises. This explains the weaker correlations between ring strength and conditioning strength for the elements support scale and inverted cross. While this explanation seems reasonable, it should be mentioned that these additional challenges differ in their severity depending on gymnasts’ individual anthropometrical characteristics.

Other reasons for the differing correlation coefficients between the different elements could be found in the test setups of the conditioning exercises compared to the requirements of the strength elements on rings. According to the work of Stone et al. [15], the transfer from conditioning exercises into the sport-specific performance strongly depends on the similarity of movement patterns (also joint angle specificity), force application, and velocity of movement. Strength elements on rings require a high level of relative maximum strength in a specific “quasi-isometric” position. With the conditioning tests, it was attempted to imitate the application of force on the rings as best as possible while eliminating coordinative aspects in order to measure the “raw strength” of the involved muscle groups. In this context, one could say that the conditioning maximum strength test for swallow/support scale was executed in a (stable) supine position. Therefore, the maximum strength of the involved muscles could be measured while isolated. Furthermore, the test was executed with 90° externally rotated arms and hands (supination), which corresponds exactly to the position of the hands when performing the element swallow on rings. In contrast, the element support scale is usually performed with a “normal” (semiprone) hand position. This may alter the application of the force on rings compared to the conditioning test and may further explain the lower correlation coefficients of the conditioning test for support scale (compared to swallow).

The conditioning maximum strength tests for inverted cross were performed in a seated position without fixation of the gymnasts’ back. This setup allowed athletes to compensate for weakness in the main muscles by using other muscles or by leaning backwards. Therefore, the conditioning strength was possibly less standardized and certainly less isolated than for the conditioning test for swallow/support scale, which could be another reason for the slightly lower correlation between conditioning maximum strength and ring-specific strength. Nevertheless, the intention was to create conditioning maximum strength tests that imitate the sensations when performing the elements on rings as best as possible. Indeed, different setups of the conditioning strength tests were explored prior to the investigation and athletes expressed the opinion unanimously that the execution felt more realistic without fixation of the back. Since strength tests must be well accepted by athletes and coaches if the aim is to apply these tests regularly in the future, this sort of feedback was crucial for deciding on the final test format.

Surprisingly, correlation coefficients were generally higher for the conditioning maximum concentric compared to the eccentric strength tests for two of the three elements. This indicates the importance of concentric conditioning maximum strength in order to hold strength elements on rings. Consequently, the development of a high level of ring-specific concentric conditioning strength should be an integral part of ring training of elite athletes. In this context, it should be mentioned that concentric strength training causes smaller joint torques compared to eccentric training [16], and therefore, fewer injuries. Furthermore, while most if not all elite athletes benefit from concentric training, the effects of eccentric training in elite athletes can differ widely [17]. In order to develop the necessary strength and stress tolerance for an entire elite athlete carrier, the ring-specific conditioning concentric strength should be the core component of the physical preparation of youth and elite gymnasts.

Nevertheless, the strongest correlation was found between the conditioning eccentric test for swallow/support scale and the element swallow. Since eccentric muscle capacities can better be assessed in an eccentric test than in a concentric test [18], this may show that holding a strength element on rings is very similar to a slow eccentric contraction (as it was supposed in a previous study [2]). However, this is only apparent with a test that meets all requirements of mechanical specificities of the element on rings. The conditioning eccentric test was performed with a very slow movement velocity (0.1 m/s) that provoked a force–time relationship similar to an isometric contraction. The maximum force was achieved at the end of the movement, which corresponds to the swallow position precisely. Therefore, the conditioning eccentric test for swallow/support scale can be considered highly valid for assessing the ring-specific strength for the element swallow. However, this was not the case for the conditioning eccentric test for inverted cross, although at the end of the eccentric movement, the position of the arms was the same as when performing the element on rings. One reason may be the previously discussed limitations (standardization). Another possible explanation for the weaker correlation was that at the moment of the investigation, none of the participating athletes were able to maintain the element inverted cross on rings with one’s own body weight. Therefore, the conditioning eccentric test may have been more unfamiliar (than the other conditioning test for swallow/support scale) and the necessary specific strength had not been developed at the same level as for the element swallow. The level of eccentric strength depends, among other factors, on the inhibition of the maximum voluntary muscle activation during eccentric contractions [19,20]. In this context, further investigations should focus on whether the relationships between conditioning strength and strength performing the element on rings is different for athletes who are able to perform the element with their own body weight. This could provide further insights into the alterations of ring-specific strength during a training process.

### 4.2. Benchmarks of Maximum Conditioning Concentric and Eccentric Strength

The highest values for the conditioning strength benchmarks were found for swallow (eccentric: 94% and concentric: 63% of body weight), and the lowest were found for inverted cross (eccentric: 70% and concentric: 56% of body weight). These results emphasize the previously mentioned varying importance of coordinative, biomechanical, and strength requirements of the different holding position, as well as the level of training of the athletes for inverted cross. The concentric conditioning strength benchmarks found in this study are comparable to another study [7] that found similar strength requirements for the element swallow (60% of body weight) with a static dumbbell exercise in supine position. In contrast, Hübner et al. [4] found higher strength benchmarks (1RM for swallow and support scale: 73% and 67% of body weight) with a traditional concentric barbell exercise. The difference (+13% of body weight) may be attributed to the greater stability and the smaller range of motion when performing the exercise with a barbell compared to dumbbells or rings.

As expected, the benchmarks for the conditioning maximum eccentric strength in order to maintain the element on rings with one’s own body weight were up to 27% higher than the benchmarks of conditioning concentric strength. The physiological mechanisms behind the higher force during eccentric compared to concentric contractions are not completely understood. However, the concentric–eccentric strength ratios found in this study are clearly lower than the values found by Hollander et al. [21], who observed from 40% (bench press) to 50% (military press) higher eccentric than concentric force under constant external load conditions for traditional upper body conditioning exercises involving similar muscle groups. In contrast to that study, the conditioning concentric test in the present study was executed with a constant load (isoinertial: 1RM) whereas the eccentric conditioning test used a constant velocity (isokinetic: 0.1 m/s). Eccentric isokinetic contractions theoretically elicit maximal muscle force over the entire range of motion. In contrast to eccentric isoinertial contractions, which induce significant accelerations, eccentric isokinetic contractions are executed with a constant movement velocity and therefore evoke smaller torques on the joints [22]. Nevertheless, due to the fully extended arms, and the resulting long lever arms, during the execution of the conditioning eccentric tests in the current study, high torques on the shoulder joint may have influenced the amount of force that could be applied. Consequently, the eccentric isokinetic test conditions reduce otherwise supra-maximal forces to a sustainable (probably near maximal) level in hopes of further reducing the risk of injury, as reported elsewhere [23].

The ratio between conditioning eccentric and concentric strength explained between 10% (inverted cross) and 32% (swallow) of the maximum strength performing the elements on rings. With regard to the generally low values of explained variance found in this context and their large differences between the elements, the ratio between conditioning concentric and eccentric strength may not be a reliable parameter in order to evaluate whether eccentric or concentric conditioning strength should be trained. Rather, it shows that concentric and eccentric strength are closely and (seemingly) individually related. Therefore, the decision of whether eccentric or concentric training should be integrated into the strength training might depend primarily on an athlete’s level of concentric strength and secondarily on the phase of preparation. Eccentric training induces high levels of stress on the joints and fatigue of the involved muscles [17], and therefore, the technical training load should be lowered during a phase of eccentric training. In order to vary training stimulus and learn to apply the conditioning strength potential in the static holding position on rings, a phase of technical training of the static element should be integrated in training after a phase of conditioning training.

**Figure 8:**
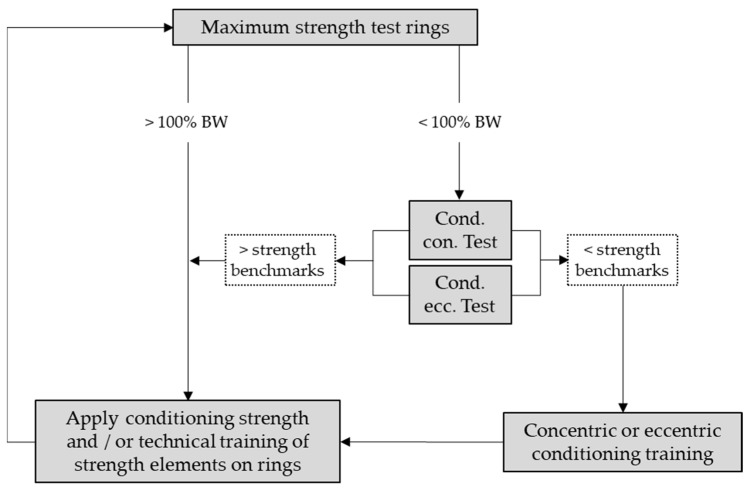
Flow chart for training recommendations. Decision tree to derive training recommendations based on the maximum strength test on rings and the conditioning (cond.), concentric (con.), and eccentric (ecc.) strength test.

Altogether, the benchmarks of conditioning concentric and eccentric strength can be used to compare an athlete’s current level of conditioning strength with the minimal strength requirements in order to maintain the strength elements on rings with his own body weight. In combination with tests of maximum strength performing the elements on rings, the conditioning strength potential and its utilization during strength hold elements on rings can be monitored, and general training recommendations may be derived with the flow chart displayed in Figure 8.

## 5. Conclusions

In conclusion, large to nearly perfect correlations were found between conditioning maximum strength tests for swallow/support scale and inverted cross and the maximum strength tests of these elements on rings (swallow > support scale > inverted cross).

The lower requirements for balance skills and compensatory forces of the element swallow, differences in mechanical specificity between the conditioning test and the element support scale, and small limitations of standardization of the conditioning test for inverted cross may explain the observed differences between the correlation coefficients.

Contrary to previous studies that developed particular tests to assess the ring-specific strength, the combination of conditioning maximum strength tests and maximum strength tests on rings in the present study may allow coaches and athletes to monitor the training process more closely. Together with the calculated benchmarks for conditioning concentric and eccentric strength for the three elements, practical training recommendations may be given that may help coaches and athletes systematize the training and focus the training either on the development of the conditioning strength or on the technical training of strength elements on rings. Consequently, future research should concentrate on the investigation of the efficacy of the different stimuli (concentric or eccentric conditioning strength, technical training) on the performance of strength elements on rings.

## Figures and Tables

**Table 1:** Mean ± standard deviation of body mass, maximum strength test (Fmax) of the elements swallow, support scale, and inverted cross on rings (body mass − counterweight or + additional weight) and the respective conditioning concentric (Fmax con) and eccentric maximum strength (Fmax ecc) on the computer-controlled device.

Element	*n*	Body Mass (kg)	Fmax Rings (kg)	Fmax con (kg)	Fmax ecc (kg)
Swallow	15	64.41 ± 4.71	55.33 ± 8.59	36.42 ± 5.00	51.91 ± 9.08
Support scale			59.10 ± 7.82		
Inverted cross	9	66.01 ± 5.03	52.68 ± 6.34	31.59 ± 5.87	42.49 ± 7.58

**Table 2:** Relationships (Pearson’s correlation: r) between maximum strength test (Fmax) of the elements swallow, support scale, and inverted cross on rings and the conditioning maximum concentric (Fmax con) and eccentric strength tests (Fmax ecc) of the two different exercises on the computer-controlled device for swallow/support scale (S, SS) and inverted cross (IC) (**: *p* < 0.01; *: *p* < 0.05).

Element	Fmax con S, SS (kg)	Fmax ecc S, SS (kg)	Fmax con IC (kg)	Fmax ecc IC (kg)
Fmax Swallow (kg)	0.87 **	0.92 **	−0.05	0.64
Fmax Support scale (kg)	0.77 **	0.65 *	−0.69	0.39
Fmax Inverted cross (kg)	0.72	0.41	0.69	0.62

**Table 3:** Benchmarks of relative (in percent of body weight: %BW) conditioning maximum concentric (con) and eccentric (ecc) strength (Fmax) in order to maintain the elements swallow (S), support scale (SS), and inverted cross (IC) with one’s own body weight (100% BW) calculated with help of the linear regression equations.

Element	Fmax con S, SS (%BW)	Fmax ecc (%BW)	Equation con	Equation ecc
Swallow 100% BW	63.05% ± 3.80%	94.10% ± 5.63%	y = 0.45x + 18.43	y = 1.09x − 15.38
Support Scale 100% BW	60.37% ± 4.95%	86.79% ± 10.59%	y = 0.45x + 15.75	y = 0.89x − 2.53
Inverted cross 100% BW	56.66% ± 6.29%	70.86 ± 8.24%	y = 0.42x + 14.44	y = 0.32x + 38.72

## Data Availability

All individual results are displayed in Table S1 (Supplementary Materials).

## Supplementary Materials

The following are available online at https://www.mdpi.com/article/10.3390/sports9060078/s1, Table S1: Individual results of all athletes of maximum strength performing strength elements on rings (swallow, support scale and inverted cross) and preconditioning maximum concentric (con) and eccentric (ecc) strength (Fmax).
