# Supplementary material for: Maximum Strength Benchmarks for Difficult Static Elements on Rings in Male Elite Gymnastics
Source: Sports (Basel). 2021 May 28;9(6):78. doi: 10.3390/sports9060078 (PMC8226549; doi:10.3390/sports9060078)
Supplement: Supplementary file 1 [file sports-09-00078-s001.zip › sports-1205924-supplementary.pdf]

sports

MDPI

Supplementary Material

Maximum Strength Benchmarks for Difficult Static Elements on Rings in Male Elite Gymnastics

Christoph Schärer *, Sarina Huber, Pascal Bucher, Claudio Capelli and Klaus Hübner

Department of Elite Sport, Swiss Federal Institute of Sport Magglingen (SFISM), 2532 Magglingen, BE, Switzerland

* Correspondence: christoph.schaerer@baspo.admin.ch; Tel.: +41-58-467-65-04

Citation: Schärer, C.; Huber, S.; Bucher, P.; Capelli, C.; Hübner, K. Maximum Strength Benchmarks for Difficult Static Elements on Rings in Male Elite Gymnastics. *Sports* **2021**, *9*, 78. https://doi.org/10.3390/sports9060078

Publisher's Note: MDPI stays neutral with regard to jurisdictional claims in published maps and institutional affiliations.

Copyright: © 2021 by the authors. Licensee MDPI, Basel, Switzerland. This article is an open access article distributed under the terms and conditions of the Creative Commons Attribution (CC BY) license (http://creativecommons.org/licenses/by/4.0/).

Table S1. Individual results of all athletes of maximum strength performing strength elements on rings (swallow, support scale and inverted cross) and preconditioning maximum concentric (con) and eccentric (ecc) strength (Fmax).

Athlete	Swallow/Support Scale					Inverted Cross			
	Body Mass	Swallow (kg)	Support scale (kg)	Fmax ecc (kg)	Fmax con (kg)	Body mass	Inverted cross (kg)	Fmax ecc (kg)	Fmax con (kg]
1	66.20	53.70	48.70	47.65	37.80	68.40	60.90	42.09	35.16
2	63.70	63.70	63.70	56.10	42.30	63.30	58.30		
3	58.70	38.70	46.20		31.71	61.80	41.80	42.29	31.86
4	65.10	40.10	50.10	33.25	28.38	66.00	48.50	35.99	28.61
5	66.00	58.50	61.00	55.33					
6	71.70	51.70	61.70	41.89	33.76				
7	58.10	45.60	54.35	43.79	34.10				
8	61.50	59.00	61.50	58.19	38.55	66.00	51.00	44.05	27.43
9	61.10	53.60	61.10	48.07	32.33				
10	65.00	65.00	60.00	64.96	41.07				
11	64.20	64.20	68.20	58.82	41.39				
12	61.40	57.65	61.40	44.85	34.86				
13	76.50	66.50	76.50	63.71	46.28				
14	62.60	62.60	62.60	62.49	37.61				
15	64.40	49.40	49.40	47.65	29.66				
16	74.30					74.30	61.80	60.58	44.46
17	58.60					58.60	48.60	38.76	29.85
18	63.30					63.30	48.30	33.92	23.36
19	72.40					72.40	54.90	42.28	31.97
